# 934. Epidemiology and Outcomes of Epstein-Barr Virus DNAemia in Adult Solid Organ Transplant Recipients

**DOI:** 10.1093/ofid/ofab466.1129

**Published:** 2021-12-04

**Authors:** Sara W Dong, Barbra M Blair, Carolyn D Alonso

**Affiliations:** Beth Israel Deaconess Medical Center, Boston, MA

## Abstract

**Background:**

Immunosuppression in solid organ transplantation (SOT) increases the risk of Epstein-Barr virus (EBV) DNAemia, which may herald development of post-transplant lymphoproliferative disease (PTLD). The objective of this study was to characterize the epidemiology and risk factors for EBV DNAemia and to describe the impact of these factors on development of PTLD in a cohort of SOT recipients.

**Methods:**

We retrospectively examined adult (≥18 years) SOT recipients between 1/1/2015 to 12/31/2019. Demographics, immunosuppression, and clinical outcomes were examined. Subjects were stratified as having a positive (EBV viral load > 200 copies/mL) or negative viral load. Sustained EBV DNAemia was defined as EBV DNA detection ( > 200 copies/mL) on at least 3 consecutive samples. Categorical variables were analyzed using chi-square or Fishers exact test. Mann-U Whitney test was used for continuous variables.

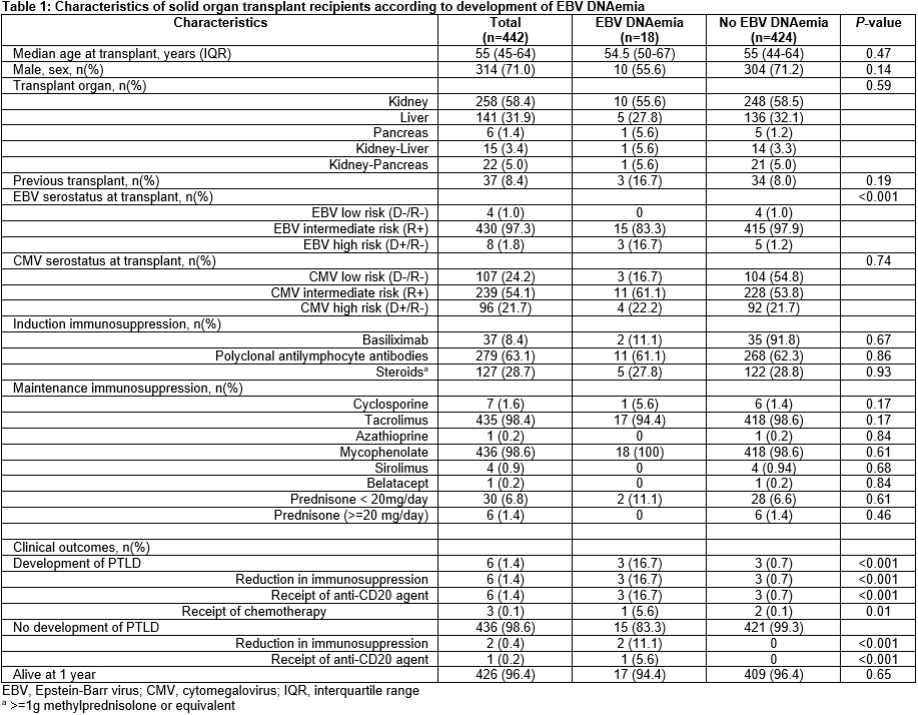

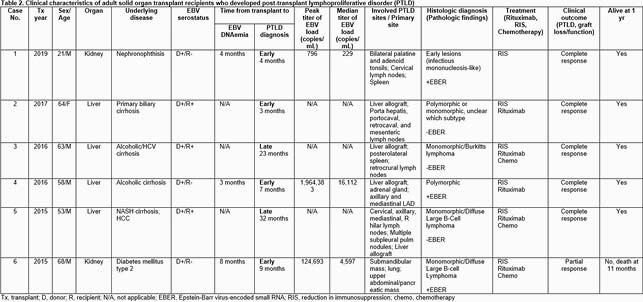

**Results:**

442 SOT recipients (258 kidney, 141 liver, 22 kidney-pancreas (KP), 15 kidney-liver, 6 pancreas) were examined. Most subjects (430, 97%) were EBV intermediate risk (recipient (R)+). 8 subjects (2%) were EBV high risk (donor (D)+, R-) and 4 (1%) were EBV low risk (D-/R-). EBV viral loads were obtained in 177/442 (40.0%) recipients. DNAemia was detected in 18/442 subjects (4.1%). It was most common in pancreas recipients(1/6; 16.7%) compared with kidney (10/258; 3.9%), liver (5/141; 3.5%), and KP recipients (1/22; 4.5%). DNAemia was most frequently observed in the D+/R- (3/8; 37.5%) group compared to intermediate risk (R+) (15/430; 3.5%) and D-/R- (0%) groups. Median time to EBV viral load detection was 14 months (range 3-60). In univariate analysis, EBV high-risk serostatus was the factor most strongly associated with development of EBV DNAemia (p< 0.001). Sustained DNAemia (median viral load 1829 copies/mL; peak viral load 1.9 million copies/mL) was observed in 8 subjects (1.8%). PTLD developed in 6 subjects (1.4%); 50% had sustained DNAemia prior to diagnosis.

**Conclusion:**

While uncommon, development of sustained EBV DNAemia was associated with subsequent development of PTLD in our cohort of adult SOT recipients. These data provide guidance for identifying subjects at risk for PTLD above and beyond baseline EBV high-risk serostatus.

**Disclosures:**

**All Authors**: No reported disclosures

